# Plasticity of the histamine H_3_ receptors after acute vestibular lesion in the adult cat

**DOI:** 10.3389/fnint.2013.00087

**Published:** 2014-01-03

**Authors:** Brahim Tighilet, Christiane Mourre, Michel Lacour

**Affiliations:** ^1^Laboratoire de Neurosciences Intégratives et Adaptatives, UMR 7260, FR - Comportement, Cerveau, Cognition (Behavior, Brain, and Cognition), Centre Saint-Charles, Case B, Centre National de la Recherche Scientifique, Aix-Marseille UniversitéMarseille, France; ^2^Laboratoire de Neurosciences Cognitives, UMR 7291, Centre Saint-Charles, Centre National de la Recherche Scientifique, Aix-Marseille UniversitéMarseille, France

**Keywords:** histamine H_3_ receptor, unilateral vestibular neurectomy, vestibular compensation, vestibular nuclei, tuberomammillary nuclei, inferior olive, solitary nucleus, cat

## Abstract

After unilateral vestibular neurectomy (UVN) many molecular and neurochemical mechanisms underlie the neurophysiological reorganizations occurring in the vestibular nuclei (VN) complex, as well as the behavioral recovery process. As a key regulator, the histaminergic system appears to be a likely candidate because drugs interfering with histamine (HA) neurotransmission facilitate behavioral recovery after vestibular lesion. This study aimed at analyzing the post-lesion changes of the histaminergic system by quantifying binding to histamine H_3_ receptors (H_3_R; mediating namely histamine autoinhibition) using a histamine H_3_ receptor agonist ([^3^H]N-α-methylhistamine). Experiments were done in brain sections of control cats (*N* = 6) and cats submitted to UVN and killed 1 (*N* = 6) or 3 (*N* = 6) weeks after the lesion. UVN induced a bilateral decrease in binding density of the agonist [^3^H]N-α-methylhistamine to H_3_R in the tuberomammillary nuclei (TMN) at 1 week post-lesion, with a predominant down-regulation in the ipsilateral TMN. The bilateral decrease remained at the 3 weeks survival time and became symmetric. Concerning brainstem structures, binding density in the VN, the prepositus hypoglossi, the subdivisions of the inferior olive decreased unilaterally on the ipsilateral side at 1 week and bilaterally 3 weeks after UVN. Similar changes were observed in the subdivisions of the solitary nucleus only 1 week after the lesion. These findings indicate vestibular lesion induces plasticity of the histamine H_3_R, which could contribute to vestibular function recovery.

## INTRODUCTION

Unilateral lesion of the peripheral vestibular system induces a syndrome of static oculomotor (nystagmus) and postural (head roll- and yaw-tilt, asymmetric extensor tone in the limb and axial muscles, increase of the surface delimited by the four legs) disorders that subside quite rapidly (few days or weeks) in a process of behavioral recovery known as vestibular compensation. This unilateral vestibular damage induces also dynamic symptoms such as vestibulo-ocular reflex gain deficit toward the lesioned side that are however long-lasting or remain relatively uncompensated. The neural mechanisms underlying this vestibular function recovery has been well documented and it is admitted that the static deficits result from the asymmetric resting discharge and their compensation is associated with a rebalanced resting activity on both sides. By contrast the compensation of the dynamics symptoms involves multiple plasticity mechanisms occurring in various brain areas (Smith and Curthoys, 1989; Curthoys, 2000; Dieringer, 2003; Lacour, 2006; Paterson et al., 2006; Dutia, 2010; Lacour and Tighilet, 2010).

Neuromodulators such as histamine could influence these plasticity mechanisms and may thus contribute to the vestibular recovery process. Numerous basic and pharmacological studies in intact and vestibular-lesioned animals, as well as in humans, put forward a link between brain histamine (HA), vestibular function and its recovery after vestibular damage. Indeed, the central histaminergic system is involved in the regulation of vestibular functions and their recovery after vestibular lesion (Bergquist and Dutia, 2006; Haas et al., 2008; Lacour and Tighilet, 2010). Histamine is highly implicated with the arousal level (Brown et al., 2001; Haas et al., 2008) and it has been shown that the horizontal vestibulo-ocular-reflex gain was very sensitive to the state of alertness (Flandrin et al., 1979; Matta and Enticott, 2004). Stimulation of the vestibular nerve enhances HA release in the hypothalamus and brainstem (Takeda et al., 1986; Horii et al., 1993, 1996; Uno et al., 1997). *In vitro* intracellular recordings from neurons in the medial and the lateral vestibular nuclei (VN; MVN and LVN) revealed HA induced depolarization via postsynaptic histamine H_1_ (H_1_R; Inverarity et al., 1993) or H_2_ receptors (H_2_R; Phelan et al., 1990; Serafin et al., 1993; Wang and Dutia, 1995; Zhang et al., 2008, 2013; Zhuang et al., 2013). Similar findings were observed in neurons in the inferior VN (IVN; Peng et al., 2013). Recent anatomical data have strengthened the histaminergic influence on vestibular functions. Indeed, HA and histidine decarboxylase (HDC) immunoreactive neurons are located exclusively in the tuberomammillary nucleus (TMN) of the hypothalamus (Panula et al., 1984; Watanabe et al., 1984; Pollard and Schwartz, 1987; Airaksinen et al., 1992; Tighilet and Lacour, 1996) and project bilaterally in various regions of the brain (Schwartz et al., 1991), including the VN (Takeda et al., 1987; Airaksinen and Panula, 1988; Panula et al., 1989). We showed that these histaminergic fibers were sparsely distributed in the whole VN complex of the cat, with a significantly higher density in the MVN and superior VN (SVN) than in the LVN and IVN (Tighilet and Lacour, 1996). The VN complexes contain all types of HA receptors (H_1_R, H_2_R, and H_3_R), as shown with ligand-binding (Bouthenet et al., 1988; Vizuete et al., 1997; Tighilet et al., 2002, 2006, 2007), *in situ* hybridization methods (Ruat et al., 1991; Vizuete et al., 1997; Pillot et al., 2002), and behavioral investigations using VN perfusion with HA receptor ligands (de Waele et al., 1992; Yabe et al., 1993). Local perfusion of the VN on one side with H_2_R antagonists or H_3_R agonists induces a stereotyped postural and oculomotor syndrome in the guinea pig that mimics that observed after labyrinthectomy (Yabe et al., 1993). In addition, we have showed that vestibular compensation in the cat was strongly accelerated under treatment with H_3_R antagonists (betahistine and thioperamide) and that H_3_R antagonists induced long-term changes in the expression of HDC mRNA in the TMN and H_3_R binding in the VN (Tighilet et al., 1995, 2006, 2007). We have postulated that release of HA likely restores the balance in neuronal activity in the VN cells on both sides, a key mechanism known to promote the vestibular compensation. Finally, histaminergic drugs are widely prescribed for treatment of vertigo and vestibular disorders (Takeda et al., 1986; Fischer, 1991; Redon et al., 2011), suggesting also that HA interferes with the vestibular system and the recovery after a vestibular loss.

The aim of this study was to analyze the plasticity of the histamine H_3_R after unilateral vestibular neurectomy (UVN). Since this receptor is the target for HA drugs favoring vestibular compensation (Tighilet et al., 2002), we analyzed the changes in histamine H_3_R density in brain networks involved in vestibular function such the VN complex, the TMN, the inferior olive (IO) complex and the solitary nucleus (SN). In addition, we performed [^3^H]N-α-methylhistamine binding to analyze the affinity of the histamine H_3_R for this ligand in control and UVN cats.

## MATERIALS AND METHODS

### ANIMALS

Experiments were performed on 18 adult domestic cats (3–5 kg) obtained from the “Centre d’élevage du Contigné” (Contigné, France). All experiments were carried out in line with the Animals (scientific procedures) Act, 1986 and associated guidelines, the European Communities Council Directive of 24 November 1986 (86/609/EEC), and the National Institutes of Health guide for the care and use of laboratory animals (NIH publications No. 8023, revised 1978). Every attempt was made to minimize both the number and the suffering of animals used in this experiment. Cats were housed in a large confined space with normal diurnal light variations and free access to water and food. Twelve animals were submitted to UVN and killed at two survival times: 1 (*N* = 6) and 3 weeks (*N* = 6). Six animals were used as a control group. The survival times were selected from our previous behavioral and electrophysiological investigations in the cat, which had showed major postural deficits in acute cats (1 week) and nearly complete recovery in compensated animals (3 weeks; see Lacour et al., 1989).

### VESTIBULAR NEURECTOMY

A left side vestibular nerve section was performed under aseptic conditions through a dissecting microscope. Animals were first anesthetized with ketamine (20 mg/kg, i.m.; Rhône-Poulenc, Mérieux, France), received analgesic (Tolfédine, 0.5 ml, i.m.; Vetoquinol, Lure, France), maintained under fluothane anesthesia (2%) and were kept at physiological body temperature using a blanket. The vestibular nerve was sectioned on the left side at a post-ganglion level in order to leave the auditory division intact after mastoidectomy, partial destruction of the bony labyrinth, and surgical exposure of the internal auditory canal (see Xerri and Lacour, 1980 for more details). Animals were maintained under antibiotics for 7 days and analgesics for 3 days. The classical postural, locomotor, and oculomotor deficits displayed by the animals in the days following nerve transection were used as criteria indicating the effectiveness of the vestibular nerve lesion. Completeness of vestibular nerve section had already been assessed by histological procedures in previous studies (Lacour et al., 1976).

### TISSUE PREPARATION

Cats of each group were deeply anesthetized with ketamine dihydrochloride (20 mg/kg, i.m., Merial, Lyon, France) and killed by decapitation; after removal from the skull, their brains were cut into several blocks containing the brainstem structures (VN, IO, SN) and the posterior hypothalamic nuclei, and the blocks were rapidly frozen with CO_2_ gas. Coronal sections (10-μm-thick) were cut in a cryostat (Leica, Rueil-Malmaison, France), thawed onto “superfrost ++” glass slides (Fisher Scientific, Elancourt, France), and stored at -80°C until radioautography.

### H_3_ RECEPTOR AUTORADIOGRAPHY

The binding of [^3^H]N-α-methylhistamine (80 Ci/mmol, NEN^TM^ Life Science Products, Boston, MA, USA) to H_3_R was performed on tissue sections as previously described (Cumming et al., 1994a; Tighilet et al., 2002, 2006, 2007). The brain sections (10 μm thick from fresh frozen tissue) were incubated with 4 nM [^3^H]N-α-methylhistamine, at 4°C in a 150-mM sodium phosphate buffer, pH 7.4, containing 2 mM magnesium chloride, and 100 μM dithiothreitol (Sigma, Saint Quentin, France). The non-specific binding component was measured by adding a large excess of thioperamide (2 mM, Tocris Cookson Ltd, Bristol, UK) 30 min before adding [^3^H]N-α-methylhistamine. After 45-min incubation, the sections were rinsed three times (each wash lasting 20 s) in the same buffer at 4°C buffer, and then rinsed once in 4°C water for 3 s. The slices were dried with a stream of cold air and exposed to tritium-sensitive film ([^3^H]Hyperfilm, Amersham). After 9 months of exposure at -80°C, the films were processed in Kodak Industrex developer at room temperature for 2 min, fixed, and then washed. Azure II stained sections were used for reference.

### [^3^H]N-α-METHYLHISTAMINE BINDING ASSAYS

To analyze the affinity of the histamine H_3_R for [^3^H]N-α-methylhistamine in lesioned and control groups of cats, we performed competition experiments. Sections of hypothalamus and brainstem structures, including the VN, the prepositus hypoglossi (PH), the SN, and the IO of controls, and both 1 and 3 weeks post-lesion cats were homogenized with a Potter homogenizer in 50 mM Tris buffer at pH 7.5, and then the homogenates were centrifuged at 1000 × *g* for 5 min. Protein level was determined according to Bradford (1976).

Hypothalamus and brainstem structure homogenates (250 μg of protein) were incubated with increasing concentrations of thioperamide in the same autoradiographic binding buffer (150 mM sodium phosphate buffer, pH 7.4, containing 2 mM magnesium chloride, and 100 μM dithiothreitol) for 45 min at room temperature in presence of 4 nM of [^3^H]N-α-methylhistamine. After incubation, 250-μl aliquots were filtrated using a cell harvester, over glass fiber filters (Whatman, GF/B) pre-soaked in 0.3% polyethylenimine. The aliquots were rapidly washed three times with 4 ml of the same buffer. The radioactivity retained by the filters was counted in a beta scintillation analyzer (Packard, Meriden, CT, USA). Curves were fit to the data with Prism non-linear least squares curve-fitting program (GraphPad Software, San Diego, CA, USA). One-site fits were tested.

### DATA QUANTIFICATION

#### H_3_ receptor binding measurement

The brainstem and posterior hypothalamic nuclei were identified with Berman’s stereotaxic atlas (Berman, 1968; Berman and Jones, 1982). The analysis of IO binding to H_3_Rs has been completed in greater detail using Brodal and Kawamura’s (1980) monograph. The autoradiograms of the binding to H_3_Rs were analyzed and quantified using NIH Image software. [^3^H] Plastic standards (Amersham) were used to calibrate ^3^H concentrations. Receptor density was expressed in fmol/mg of protein and evaluated for both the brainstem structures and the TMN. A mean receptor density value was calculated for each nucleus from 60 serial sections. The specific binding value was determined as the difference between total and non-specific binding components for a given area and was evaluated as the mean ± SEM. The density of [^3^H]N-α-methylhistamine binding sites was evaluated in the following brainstem structures: each of the four main VN (MVN, IVN, SVN, and LVN, respectively), the three subdivisions of the IO (medial accessory, dorsal accessory, and principal nucleus: MIO, DIO, and PIO, respectively), the principal subdivisions of the principal nucleus of the IO [the dorsomedial cell column (DMCC), the dorsal cap (DC), the beta nucleus (β nucleus), the ventrolateral outgrowth (VLO)], the two subdivisions of the SN [lateral and medial nuclei of the solitary tract (SL and SM), PH, and the posterior hypothalamic nuclei]. These last structures included the TMN, the medial mammillary nucleus (MMN), the dorsal hypothalamic area (HDA), the lateral hypothalamic area (HLA), and the posterior hypothalamic area (HPA).

#### Statistical analysis

Analysis of variance (Super Anova) was used to test the effects of the vestibular lesion (intact versus UVN cats), the survival period (1 week versus 3 weeks), the side (deafferented versus intact), and the structure (VN, the IO and SN subdivisions, the PH, and the posterior hypothalamic nuclei) on H_3_R binding density, and to determine the interactions between these variables. Super ANOVA was followed by *post hoc* analysis with the Scheffé test and multicomparison Fisher’s test (stateview II software).

## RESULTS

All the cats that underwent a left vestibular neurectomy exhibited ocular nystagmus (fast phase directed to the right), head tilt, postural asymmetry, and falling to the left side in the first week following the lesion. Most of them recovered sufficiently in 2 or 3 days to feed by themselves. Those killed at the 3 weeks survival time had shown nearly complete behavioral recovery.

In the control cats, a relatively high [^3^H]N-α-methylhistamine binding density was found in both the TMN and brainstem nuclei. No significant differences were seen between the left and the right sides and no significant interindividual differences were found in the different groups, as shown by the analysis of variance.

H_3_Rs binding density in lesioned cats differed markedly from controls. Repeated-measure analysis of variance demonstrated that group (controls versus lesioned cats) and survival period (1 week versus 3 weeks) constituted the main fixed effects providing the sources of variation among animals. In addition, a significant group × post-lesion time was observed indicating that changes in H_3_Rs binding density overtime were different in the two groups of UVN cats.

### H_3_ RECEPTOR BINDING SITES IN THE CAT POSTERIOR HYPOTHALAMUS

The effects of UVN were examined on the density of histamine H_3_Rs in cat brain. [^3^H]N-α-methylhistamine (4 nM) was used to generate autoradiograms in brain sections in the three groups of cats. Specific binding of [^3^H]N-α-methylhistamine amounted to 70% of total binding to cat sections. Non-specific binding was homogeneous in the different regions studied.

In the posterior hypothalamus of control cats, the distribution of [^3^H]N-α-methylhistamine binding sites was heterogeneous. The highest densities (>150 fmol/mg protein) were in the TMN and the MMN. In contrast, the HLA had the lowest binding density (<100 fmol/mg protein). The HDA and the HPA contained moderate levels of binding sites.

**FIGURE 1:**
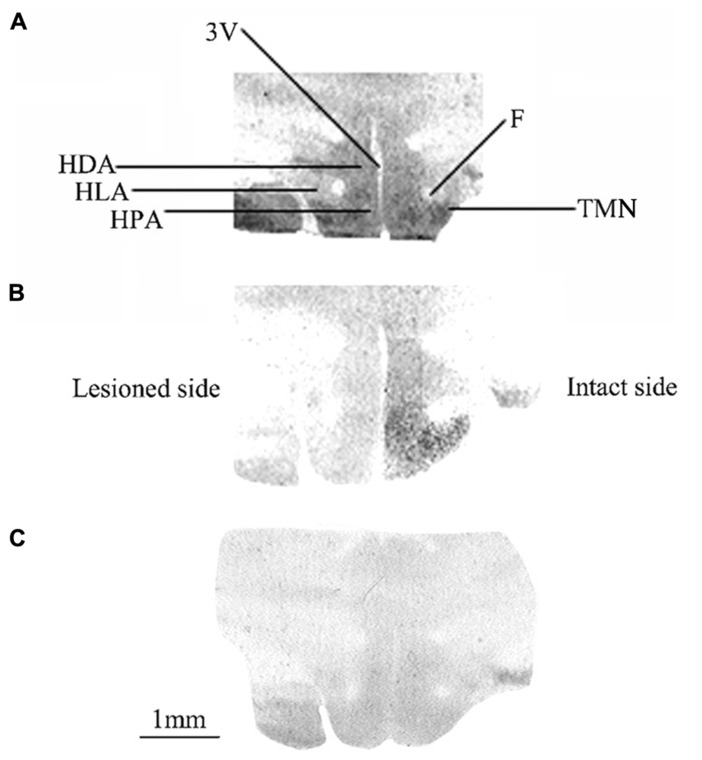
**(A–C)** [^3^H]N-α-methylhistamine binding sites in the cat posterior hypothalamus. Coronal sections from three representative control and vestibular-lesioned cats showing decrease of H_3_ receptor binding sites in the different structures of the posterior hypothalamus 1 **(B)** or 3 **(C)** weeks after unilateral vestibular neurectomy, as compared to controls **(A)**. HLA, lateral hypothalamic area; HPA, posterior hypothalamic area; HDA, dorsal hypothalamic area; F, fornix; TMN, tuberomammillary nucleus; 3V, third ventricle. Bar: 1 mm.

**Figure 1** shows typical autoradiograms of frontal sections of the posterior hypothalamus from three representative animals either unlesioned (controls: **Figure 1A**) or observed after 1 (**Figure 1B**) or 3 (**Figure 1C**) weeks after UVN. The binding of the agonist [^3^H]N-α-methylhistamine to H_3_R is shown for the controls (**Figure 1A**) as dark stained structures. A high binding density was seen in different areas including the HPA, HLA, and HDA as well as in the TMN and the MMN. Compared to the controls, the UVN induced a bilateral decrease of the binding density in all parts of the posterior hypothalamus including the TMN, with a lower level on the lesioned side compared to the intact side at 1 week post-lesion (**Figure 1B**). This bilateral decrease persisted and became symmetric 3 weeks after the lesion (**Figure 1C**).

**FIGURE 2:**
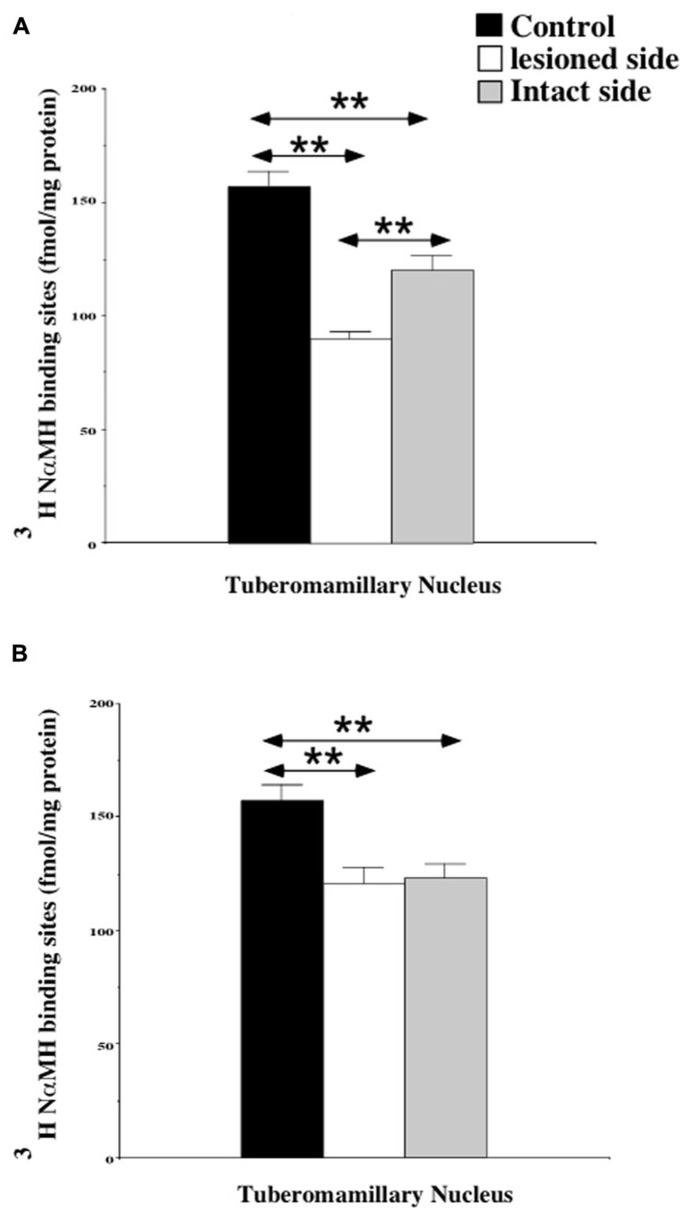
**(A,B)** Quantification of the effects of a unilateral vestibular neurectomy on the density of [^3^H]N-α-methylhistamine binding sites in the cat posterior hypothalamus. The [^3^H]N-α-methylhistamine binding to histamine H_3_ receptors is expressed in fmol/mg protein (ordinates) as the mean ± SEM. Data from the tuberomammillary nucleus (TMN) are given as the average value for the right and left structures in the controls (black histograms); they are provided separately for each side [lesioned (thick hatched histograms) versus intact (thin hatched histograms)] for the cats killed 1 **(A)** or 3 **(B)** weeks after unilateral vestibular neurectomy. **P < 0.0001.

The quantitative analysis of the [^3^H]N-α-methylhistamine binding sites densities in the TMN is shown in the **Figure 2**. The H_3_R binding density was 157.5 ± 6.3 fmol/mg of protein on average in the TMN of control cats [150.4 ± 8.6 and 164.5 ± 9.3 on the right and left sides, respectively: not statistically significant (NS)]. In the subgroup of cats examined 1 week after UVN (**Figure 2A**), the H_3_R binding density was significantly decreased in both the lesioned (90.5 ± 0.4; 42%; *P* < 0.0001) and the intact (120.6 ± 6.1; 23%; *P* < 0.0001) sides when compared to the controls. In addition, the H_3_R binding density on the lesioned side was significantly lower than that on the intact side (25%; *P* < 0.0001). In the subgroup of cats examined 3 weeks after UVN (**Figure 2B**), there was no significant difference between the intact (123.1 ± 5.8) and the lesioned (120.9 ± 7.1) sides but the [^3^H]N-α-methylhistamine binding sites densities remained significantly lower than that of the controls (22%; *P* < 0.0001 and 23%; *P* < 0.0001; for the intact and lesioned sides, respectively).

### H_3_ RECEPTOR BINDING SITES IN THE CAT BRAIN STEM

**FIGURE 3:**
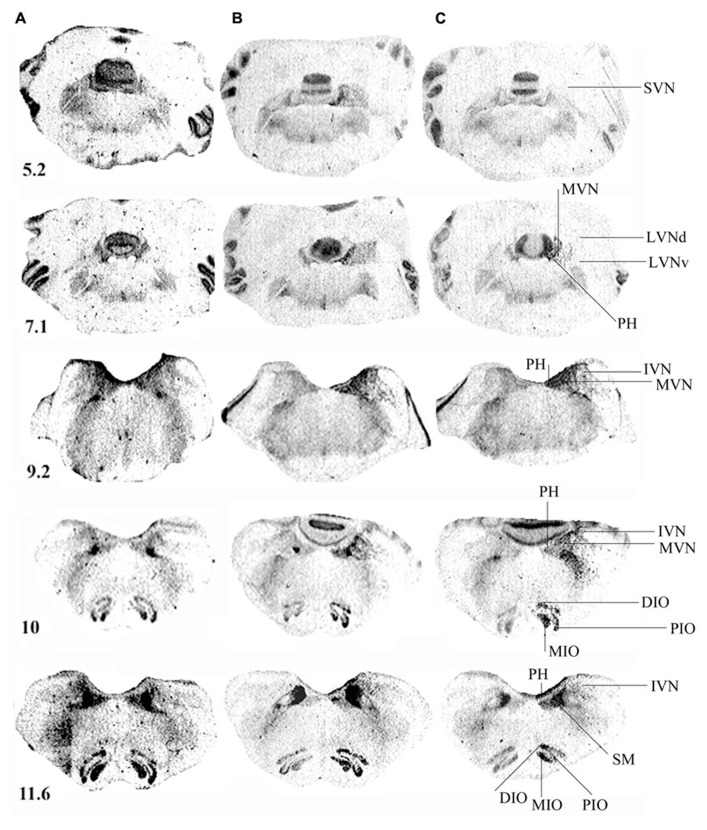
**(A–C)** [^3^H]N-α-methylhistamine binding sites in the cat brainstem. Coronal sections from three representative control and unilateral vestibular neurectomized cats showing decreases in histamine H_3_ receptor binding in the different structures of the brainstem on the lesioned side (left side in the figure) 1 **(B)** or 3 **(C)** weeks after unilateral vestibular neurectomy, as compared to the controls **(A)**. Illustrations are given for serial sections collected from the rostral (5.2) to the caudal (12.1) parts of the brainstem. IVN, inferior vestibular nucleus; LVNd and v, lateral vestibular nucleus, dorsal and ventral parts; MVN, medial vestibular nucleus; SVN, superior vestibular nucleus; PH, prepositus hypoglossi; DIO, MIO, and PIO, dorsal, medial, and posterior parts of the inferior olive, respectively; SM, medial part of the solitary tract. Bar: 1 mm.

**Figure 3** illustrates the spatial distribution of binding density in representative serial frontal sections collected from the rostral (5.2) to the caudal (11.6) parts of the brainstem in a control cat (**Figure 3A**) and in two representative cats killed 1 (**Figure 3B**) or 3 (**Figure 3C**) weeks after UVN. In the control cat, the pattern of H_3_R binding was heterogeneous: highest levels of binding sites were found in the SN complex while lower levels were found in the IO and the VN complexes.

#### Vestibular complex

The [^3^H]N-α-methylhistamine binding sites were heterogeneously distributed in the vestibular complex. Among the VN, the MVN and SVN showed the highest level of binding. The IVN showed moderate levels of binding while the lowest level was observed in the LVN. The binding density was also high in the PH nuclei (**Figures 3A–C**). Whatever the stereotaxic reference planes examined, no binding density higher than 150 fmol/mg protein (high level) was present in the vestibular complex.

**FIGURE 4:**
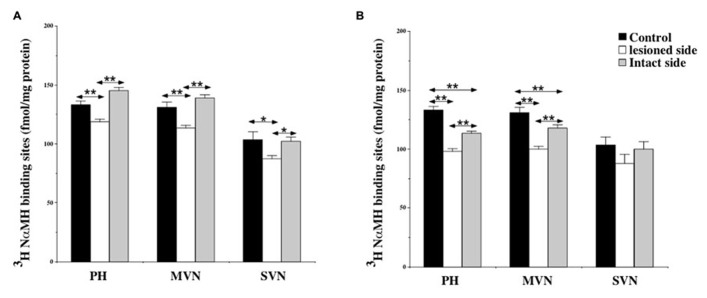
**(A,B)** Effects of a unilateral vestibular neurectomy on the density of [^3^H]N-α-methylhistamine binding sites in the vestibular nuclei and the prepositus hypoglossi. Changes in histamine H_3_ receptor binding in the vestibular nuclei and the prepositus hypoglossi 1 **(A)** or 3 **(B)** weeks after unilateral vestibular neurectomy. Quantitative analysis is expressed as mean values and standard errors of femtomole of [^3^H]N-α-methylhistamine specifically bound per milligram of protein from autoradiograms. Data from the prepositus hypoglossi (PH), and the medial (MVN) and superior (SVN) vestibular nuclei are given as the average value of the right and left structures for the controls (black histograms); they are provided separately for each side [lesioned (thick hatched histograms) versus intact (thin hatched histograms)] for the cats killed 1 **(A)** or 3 **(B)** weeks after unilateral vestibular neurectomy. *P < 0.001, **P < 0.0001.

At 7 days post-lesion, [^3^H]N-α-methylhistamine binding site density significantly decreased on the deafferented side in several nuclei relative to the controls: the PH (11%; *P* < 0.0001), the MVN (13.5%; *P* < 0.0001), and the SVN (16%; *P* < 0.001; see **Figures 3B** and **4A**). The binding remained unchanged in the other VN (LVN and IVN).

Bilateral changes in [^3^H]N-α-methylhistamine binding site density were observed in the VN complexes 21 days after UVN. The lesion induced a significant bilateral decrease with an ipsilateral predominance in the PH (26 and 14%; *P* < 0.0001) and the MVN (24 and 10%; *P* < 0.0001) on the lesioned and intact sides respectively (see **Figures 3C** and **4B**). The binding site density remained unchanged for the LVN and SVN.

#### Inferior olive complex

**Figure 3** illustrates the distribution of [^3^H]N-α-methylhistamine binding sites in the IO complex. In control cats, the binding signals were observed in all subregions but the intensity of signals varied markedly between the subregions. Moderate H_3_R binding was detected in the DMCC, the DC, and the VLO while strong binding was observed in the β nucleus, the MIO, the DIO, and the PIO.

**FIGURE 5:**
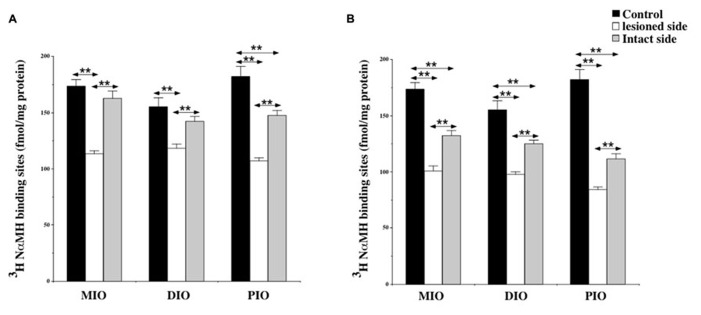
**(A,B)** Effects of a unilateral vestibular neurectomy on the density of [^3^H]N-α-methylhistamine binding sites in the three parts of the inferior olive. Changes in histamine H_3_ receptor binding in the inferior olive 1 **(A)** or 3 **(B)** weeks after unilateral vestibular neurectomy. Results are expressed as mean values and standard errors of femtomole of [^3^H]N-α-methylhistamine specifically bound per milligram of protein from autoradiograms. Data from the medial (MIO), the dorsal (DIO), and the posterior (PIO) parts of inferior olive are given as the average value of the right and left structures for the controls (black histograms); they are provided separately for each side [lesioned (thick hatched histograms) versus intact (thin hatched histograms)] for the cats killed 1 **(A)** or 3 **(B)** weeks after unilateral vestibular neurectomy. **P < 0.0001.

At 7 days post-lesion, the density of [^3^H]N-α-methylhistamine receptor binding was significantly lower on the ispsilateral side compared to the controls and the contralateral side in the MIO (35 and 31%; *P* < 0.0001, respectively), the DIO (23 and 17%; *P* < 0.0001), and the PIO (41 and 28%; *P* < 0.0001). In contrast, binding sites densities in the IO nuclei on the contralateral side were unchanged, except that the PIO showed lower values (19%; *P* < 0.0001; **Figure 5A**).

As shown for the VN, bilateral changes in [^3^H]N-α-methylhistamine binding site density were detected in the three subdivisions of the IO 21 days after UVN. The lesion induced a significant bilateral decrease in the MIO (42 and 27%; *P* < 0.0001), the DIO (38 and 20%; *P* < 0.0001) and the PIO (58 and 40%; *P* < 0.0001) on the lesioned and intact sides, respectively (**Figure 5B**). The lesioned side was more greatly reduced than the intact side (24%; *P* < 0.0001; 22%; *P* < 0.0001, and 25%; *P* < 0.0001 for the MIO, DIO, and the PIO, respectively).

**FIGURE 6:**
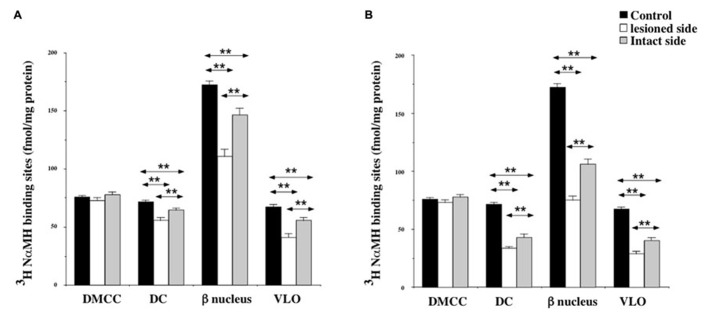
**(A,B)** Effects of a unilateral vestibular neurectomy on the density of [^3^H]N-α-methylhistamine binding sites in the different subregions of the PIO. Changes in histamine H_3_ receptor binding in the different subregions of the PIO 1 **(A)** or 3 **(B)** weeks after unilateral vestibular neurectomy. Results are expressed as mean values and standard errors of femtomole of [^3^H]N-α-methylhistamine specifically bound per milligram of protein from autoradiograms. Data from the dorsomedial cell column (DMCC), the dorsal cap (DC), the beta nucleus (b nucleus) and the ventrolateral outgrowth (VLO) are given as the average value of the right and left structures for the controls (black histograms); they are provided separately for each side [lesioned (thick hatched histograms) versus intact (thin hatched histograms)] for the cats killed 1 **(A)** or 3 **(B)** weeks after unilateral vestibular neurectomy. **P < 0.0001.

Except for the DMCC which remained unaffected by the vestibular lesion, bilateral changes in [^3^H]N-α-methylhistamine binding site density were detected in the main subdivisions of the PIO (DC, VLO and β nucleus) 1 and 3 weeks after UVN. In addition, these later subdivisions, showed a significant decrease on the lesioned side when compared to the intact side at the two survival periods (**Figures 6A,B**).

#### Solitary nucleus

Among the structures analyzed in this study, the SN showed the highest H_3_ binding density. The binding was about 400 fmol/mg protein in the SM and 100 fmol/mg protein in the SL.

**FIGURE 7:**
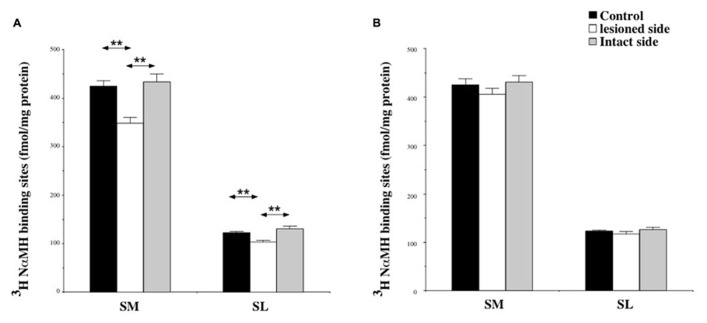
**(A,B)** Effects of a unilateral vestibular neurectomy on the density of [^3^H]N-α-methylhistamine binding sites in the two subdivisions of the solitary nucleus complex. Changes in histamine H_3_ receptor binding in the two subdivisions of the SN 1 **(A)** or 3 **(B)** weeks after unilateral vestibular neurectomy. Results are expressed as mean values and standard errors of femtomole of [^3^H]N-α-methylhistamine specifically bound per milligram of protein from autoradiograms. Data from the lateral nucleus of the solitary tract (SL), and the medial nucleus of the solitary tract (SM) are given as the average value of the right and left structures for the controls (black histograms); they are provided separately for each side [lesioned (thick hatched histograms) versus intact (thin hatched histograms)] for the cats killed 1 **(A)** or 3 **(B)** weeks after unilateral vestibular neurectomy. **P < 0.0001.

At 7 days post-lesion, the density of [^3^H]N-α-methylhistamine receptor binding was significantly lower on the ispsilateral side (**Figure 7A**) compared to the controls and the contralateral side in the SM (22 and 24%; *P* < 0.0001, respectively) and the SL (18 and 25%; *P* < 0.0001, respectively). The binding regained control values 3 weeks after the lesion (**Figure 7B**).

### COMPETITION BETWEEN [^3^H]N-α-METHYLHISTAMINE AND THIOPERAMIDE ON HOMOGENATES OF CONTROL AND VESTIBULAR-LESIONED CAT HYPOTHALAMUS AND BRAINSTEM

**Table 1** shows the specific binding of [^3^H]N-α-methylhistamine (4 nM) in the hypothalamus and brainstem structure homogenates of the three groups of cats. As shown previously for the TMN (Tighilet et al., 2002), [^3^H]N-α-methylhistamine binding was significantly (*P* < 0.05) reduced bilaterally in the hypothalamus at 1 week post-lesion, with a predominant down-regulation in the lesioned side (28%) compared to the intact side (26%). A significant bilateral and symmetric reduction was observed at 3 weeks post-lesion (28 and 31% in the intact and lesioned sides, respectively, *P* < 0.05).

**Table 1:** [^**3**^H]N-α-methylhistamine binding in the lesioned versus control cats.

Cat group	Mean ± SEM	
	1 Week	3 Weeks
**Hypothalamus**
Control	41.65 ± 1.38	
Intact side	41.66 ± 1.35	39.84 ± 1.14
Lesioned side	29.96^*^^a^ ± 1.52	27.81^*^^a^ ± 1.86
**Brainstem**
Control	45.39 ± 1.73	
Intact side	45.78 ± 1.56	36.02^a^ ± 0.75
Lesioned side	32.08^*^^a^ ± 0.33	25.91^*^^a^ ± 0.44

*[^3^H]N-α-methylhistamine (4 nM) was incubated with hypothalamus and brainstem homogenates (250 μg protein) in the same autoradiography binding buffer for each group of cats. The [^3^H]N-α-methylhistamine binding is expressed in fmol/mg of protein.*

* *P* < 0.05, Student's *t*-test, comparison with intact side.

a *P* < 0.05, Student's *t*-test, comparison with control animals.

For the brainstem, binding on the lesioned side was significantly lower than on the intact side 1 week and 3 weeks after the lesion (30 and 29%, respectively, *P* < 0.05). In comparison with the controls, the binding was significantly decreased on the lesioned side only at 1 week (30%, *P* < 0.05); it was significantly reduced for both lesioned and intact sides at 3 weeks (43 and 21%, *P* < 0.05). The reductions were in the same range as that observed in the autoradiographic study.

**FIGURE 8:**
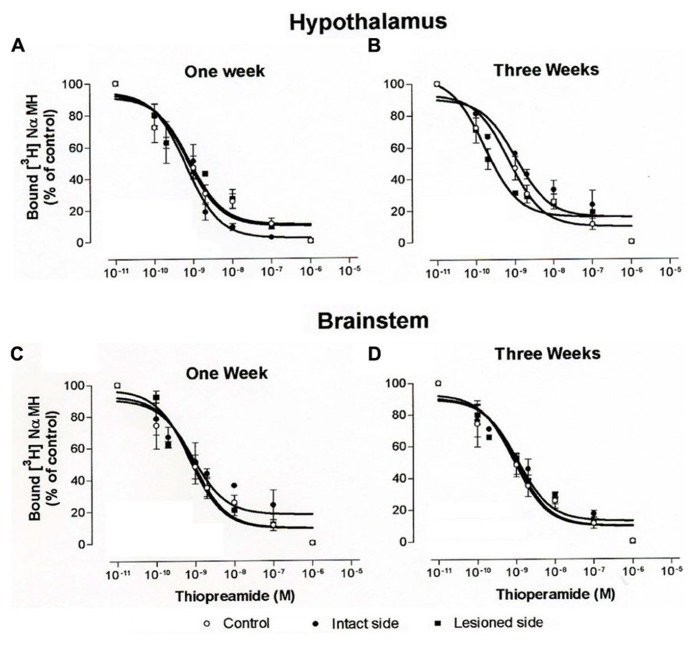
**(A–D)**. Binding of [^3^H]N-α-methylhistamine. Competition curves obtained from binding experiments involving [^3^H]N-α-methylhistamine and increasing concentrations of thioperamide in hypothalamus **(A,B)** and brainstem **(C,D)** homogenates from control cats and vestibular-lesioned cats killed 1 **(A,C)** and 3 **(B,D)** weeks after lesion.

Increasing concentrations of thioperamide gradually inhibited [^3^H]N-α-methylhistamine specific binding in hypothalamus (**Figures 8A,B**) and brainstem homogenates (**Figures 8C,D**). Under our binding conditions, similar to those of the autoradiographic procedures with the presence of sodium ions, the concentration of thioperamide inducing 50% inhibition of [^3^H]N-α-methylhistamine binding (IC_50_) was 1.92 ± 1.65 and 1.00 ± 1.56 nM in control cats (*N* = 3) for the hypothalamus and the brainstem, respectively. The IC_50_ in the cats 1 week after vestibular lesion (*N* = 3) were 0.85 ± 1.41 (intact side) and 4.5 ± 1.37 (lesioned side) for the hypothalamus, and 0.98 ± 1.61 (intact side) and 0.3 ± 1.38 (lesioned side) for the brainstem. Three weeks after lesion, the IC_50_ of cats (*N* = 3) were 1.14 ± 1.5 and 1.67 ± 1.71 for the ipsilateral and contralateral hypothalamus, respectively, and 0.66 ± 1.61 and 2.13 ± 1.82 for the brainstem on the intact and lesioned sides, respectively. The statistical analysis on these IC_50_ values showed no changes in the affinity of thioperamide for H_3_Rs in competition with [^3^H]N-α-methylhistamine, i.e., no change in IC_50_ value of the radioligand whatever the groups of cats.

## DISCUSSION

Unilateral vestibular neurectomy induced a bilateral decrease in binding of the agonist [^3^H]N-α-methylhistamine to H_3_R in the TMN at 1 week post-lesion, with a predominant down-regulation in the ipsilateral TMN. The bilateral decrease remained at the 3 weeks survival time and became symmetric. Concerning brainstem structures, N-α-methylhistamine binding in the VN, the PH and the different subdivisions of the IO decreased unilaterally on the ipsilateral side at 1 week and bilaterally 3 weeks after UVN with an ipsilateral predominance. Similar changes were observed in the subdivisions of the SN only 1 week after the lesion. These findings indicate a vestibular lesion-induced plasticity of the H_3_Rs, which could contribute to vestibular function recovery.

### DISTRIBUTION OF THE H_3_Rs BINDING SITES

The distribution of H_3_Rs binding evidenced by autoradiographic studies in various species (rat, Cumming et al., 1991; Pollard et al., 1993; Anichtchik et al., 2000; guinea pig, Cumming et al., 1994b; mouse, Cumming et al., 1994b; Janssen et al., 2000; primate and human, West et al., 1999) showed a wide and heterogeneous distribution of H_3_Rs binding sites in various brain areas (cerebral cortex, hippocampal formation, hypothalamus, TMN,… and lower brain stem areas: for review, see Schwartz et al., 1991). Our autoradiographic investigation confirms this heterogeneous distribution in the cat brain stem (IO, PH, VN, SN) and diencephalon (posterior hypothalamus). The H_3_Rs binding distribution reflects the distribution of histaminergic nerve terminals in the VN complex, with H_3_Rs binding site density higher in the MVN and the SVN than in the LVN and IVN (Tighilet and Lacour, 1996). However, the HA-labeled fibers and varicosities in the VN were much less dense than in the TMN (Tighilet and Lacour, 1996, 1997), while the density of the H_3_Rs binding sites was similar in these structures. This finding suggests that the H_3_Rs in the VN are composed of both auto and heteroreceptors and that the H_3_ heteroreceptors located on non-histaminergic afferents or on vestibular perikarya, as recently shown by Pillot et al. (2002) would predominate.

### EFFECTS OF UNILATERAL VESTIBULAR LESION ON H_3_Rs BINDING SITES

The UVN produced a significant reduction in [^3^H]N-α-methylhistamine binding in the VN, the PH as well as the different subdivisions of the IO and the SN. This reduction was observed only on the deafferented side at 1 week but bilaterally 3 weeks after UVN, with an ipsilateral predominance. It could be caused by a down-regulation of the histamine H_3_Rs or a change in the affinity of the radioligand for the H_3_Rs. Our findings in competition studies with thioperamide did not show significant changes in IC_50_ values of the [^3^H]N-α-methylhistamine radioligand to H_3_Rs in UVN cats, strengthening the hypothesis of a down-regulation of the histamine H_3_Rs.

Several hypotheses can explain this down-regulation. The first concerns the presynaptic histamine H_3_ autoreceptors and heteroreceptors in the VN complex. The changes found 1 week after UVN can be explained by the bilateral anatomical connections between the VN complexes and the posterior hypothalamus strengthening the idea of vestibulo-hypothalamic loop activation due to VNC electrical asymmetry. Indeed, the MVN project bilaterally to the posterior hypothalamus (Ericson et al., 1991) but direct and predominantly contralateral projections from the MVN to the HPA have been found in the monkey (Matsuyama et al., 1996). While the posterior hypothalamus sends histaminergic fibers to the ipsilateral MVN (Pollard and Schwartz, 1987). The asymmetrical firing rate of the VN cells in acute UVN cats (1 week), with reduced activity on the lesioned side and increased activity on the intact side for both the MVN (Precht et al., 1966) and the LVN (Zennou-Azogui et al., 1993) can therefore account for the HDC mRNA up-regulation, particularly pronounced in the TMN at 1 week post-lesion on the lesioned side (Tighilet et al., 2006). The time-course of HDC mRNA expression in the TMN of the UVN cats correlates with electrophysiological data. Electrophysiological investigations in the UVN cat still revealed, 3 weeks post-lesion, asymmetrical spontaneous firing rates between the bilateral VNCs, but the imbalance was attenuated. This attenuated imbalance may account for the lower asymmetry in HDC mRNA expression observed between the two TMN at this stage (Tighilet et al., 2006, 2007). Therefore, and as previously discussed (Tighilet et al., 2002), the autoradiographic [^3^H]N-α-methylhistamine binding reduction very likely results from a down-regulation of the histamine H_3_ receptors. One week after UVN, the high level of histamine synthesis in the ipsilateral TMN (Tighilet et al., 2006) and release in the ipsilateral VN (Tighilet and Lacour, 1997) due to vestibular lesion very likely leads to a high desensitization of the histamine H_3_ receptor, its internalization and degradation in the deafferented VN. Based on the data obtained by *in situ* hybridization and electrophysiology, this reduction was observed bilaterally 3 weeks after UVN, with an ipsilateral predominance in the same brainstem structures. Such molecular mechanisms have been demonstrated in the guinea pig ileum for the histamine H_3_R (Perez-Garcia et al., 1998) and in a specific cell line for the histamine H_2_R (Fukushima et al., 1997).

Activity-dependent plasticity is a second hypothesis accounting for the H_3_Rs binding asymmetries. As reported above, the second-order vestibular neurons on the deafferented side (type I) lose their major excitatory input after UVN and become silent, while those on the intact side show a slightly increased resting discharge. The H_3_Rs down-regulation at 1 and 3 weeks post-lesion could result from this decreased activity, at least on the lesioned side since receptors expression can be activity dependent (Tighilet et al., 1998). This cannot, however, explain the binding sites reduction seen contralaterally at 3 weeks since VN activity on the intact side is near normal at least for the type I neurons. The effect observed on the contralateral VN may mainly concern other populations of VN cells like GABA interneurons (Tighilet and Lacour, 2001). A third hypothesis could be that the primary vestibular afferents, which constitute the vestibular nerve, could carry in their terminals H_3_ receptors that disappear with the degenerative fibers induced by the nerve section. In line with this hypothesis, recent data reported the presence of H_3_ receptors in neurons of mouse Scarpa’s ganglion (Tritto et al., 2009).

The H_3_Rs binding site density was roughly similarly modified in all part of the posterior hypothalamus including the TMN and the subdivisions of the IO, with ipsilateral and bilateral binding reductions at 1 and 3 weeks after UVN, respectively. Concerning the SN, the binding decrease was observed exclusively on the lesioned side at 1 week after UVN. Mechanisms similar to the first hypothesis postulated below for the VN are the most appropriate to interpret this result since HA-like immunoreactive afferent fibers were found in the TMN (Tighilet and Lacour, 1996), the SN and IO complexes in the cat (unpublished data) and the rabbit IO (Iwase et al., 1993).

However, our results are in conflict with a recent report describing the changes in histamine H_1_, H_2_, and H_3_ receptors expression in the rat MVN and flocculus after unilateral labyrinthectomy (UL; Zhou et al., 2013). Using quantitative real-time PCR, western blotting and immunohistochemistry, these authors showed an up-regulation of all HA receptors on the first and third day after UL in the ipsilesional flocculus, and on the first day in the ipsilesional MVN compared to the sham controls as well as the contralateral side. The mRNA and protein levels of H_1_, H_2_, and H_3_ receptors returned to basal levels at 3 days (MVN) and 7 days (flocculus) after UL. By performing *in situ* hybridization in UL rats, Lozada et al. (2004) found also an increase in the mRNA levels of H_3_ receptor isoforms in the MVN on the first day after UL. Such discrepancy between the data might be due first to the surgical approach. We have demonstrated that the recovery mechanisms and the cellular plastic events occurring in the VN are different after UL and UVN in the cat model (Lacour et al., 2009; Dutheil et al., 2011). It might depend also on the animal species tested. Indeed, the temporal changes in the static vestibular deficits are different in the rat (behavioral recovery achieved within 1 week) compared to the cat (behavioral recovery requires a longer time period: 6 weeks).

### HISTAMINE H_3_ RECEPTORS PLASTICITY, VESTIBULAR COMPENSATION, AND PHARMACOLOGICAL IMPLICATIONS

The interesting point of this investigation is the functional role of such H_3_R plasticity in the vestibular compensation. It is well established that the restoration of vestibular functions is subtended by a physiological model involving restoration of balanced electrical activity between homologous VN. Does the H_3_R plasticity constitute a neurochemical mechanism involved in the recovery of a balanced electric activity between homologus VN?

The H_3_ receptor binding asymmetries observed in the VN and in the PH in the acute stage of vestibular compensation (7 days) are correlated with those seen behaviorally and electrophysiologically (Smith and Curthoys, 1989) at this time. These asymmetries persist at the compensated stage (3 weeks) in the MVN and the PH, but these nuclei exhibited also a significant bilateral decrease compared to the controls. The H_3_Rs binding changes observed in the MVN and the PH 3 weeks after UVN can be seen as a long-term plastic change involved in regulating sensitivity of the second-order vestibular cells on both sides with a higher effect on the lesioned side. Intracellular recordings from neurons in the MVN have revealed several classes of neurons, all of which are depolarized by histamine via an action at postsynaptic H_1_ (Inverarity et al., 1993) or H_2_ receptors (Phelan et al., 1990; Serafin et al., 1993; Wang and Dutia, 1995). If we consider the presynaptic H_3_ autoreceptors located on histaminergic terminals innervating the VN, particularly the MVN (Takeda et al., 1987; Steinbusch, 1991; Tighilet and Lacour, 1996), their bilateral down-regulation observed in the MVN at 3 weeks could produce an increase in histamine synthesis and release in this nucleus on both sides, contributing to rebalance the bilateral activity and thus favoring the behavioral recovery process. Interestingly, recent data using H_3_ receptor gene transcript have demonstrated the presence of high levels of H_3_ receptors mRNA on vestibular perikarya themselves including the MVN (Pillot et al., 2002). As postulated by these authors, besides autoreceptors, these H_3_ receptors may explain that systemic administration of H_3_ receptor antagonists or inverse agonists strongly decrease the horizontal vestibular-ocular reflex in the guinea pig (Yabe et al., 1993) and facilitate vestibular compensation in the cat (Tighilet et al., 1995), thereby suggesting the potential interest of these compounds as anti-vertigo drugs.

### H_3_ RECEPTORS, NEUROTRANSMISSION, AND VESTIBULAR COMPENSATION

Since the original demonstration by Arrang et al. (1983) that histamine H_3_ receptors inhibit histamine synthesis and release, histamine has been found to inhibit the release of many other transmitters via this receptor, including glutamate (Brown and Reymann, 1996), GABA (Jang et al., 2001), noradrenaline (Schlicker et al., 1989), dopamine (Schlicker et al., 1993), acetylcholine (Arrang et al., 1995), serotonin (Schlicker et al., 1988), and various peptides (Hill et al., 1997). Interestingly, these different classes of neurotransmitters are present in the VN and are involved in both vestibular functions and vestibular compensation (de Waele et al., 1995). Let us consider the GABAergic system, the glutamatergic system and the H_3_ receptors location in the MVN: (1) on histaminergic fibers or other afferents fibers innervating the MVN. (2) On terminals of the inhibitory interneurons in the MVN that make synaptic contacts on second-order excitatory neurons. (3) On the terminals of second-order excitatory MVN neurons making cross-commissural synaptic contacts on contralateral MVN inhibitory interneurons. After UVN, down-regulation of the H_3_ receptors in the MVN could facilitate GABA release from cerebellar inputs and from inhibitory interneurons that make synaptic contacts with second-order neurons, or facilitate glutamate release from terminals of second-order MVN neurons that synapse on inhibitory interneurons in the contralateral MVN. Modulation of GABAergic and glutamatergic by the H_3_R should restore the balance between the VN on both sides.

### H_3_ RECEPTOR PLASTICITY IN THE TMN

Unilateral vestibular neurectomy induced an up-regulation of HDC mRNA expression in the TMN resulting from an activation of a vestibulo-hypothalamo-vestibular loop. The mechanism of action of histamine on to the VN helps to explain the functional role of this neural loop activated when asymmetrical inputs reach the central vestibular structures (Horii et al., 1993). This loop could convey signals that promote the regulation of HDC gene expression leading to the release of histamine in the VN. The modulatory action of histamine could intervene in rebalancing the activity between homologous VN to facilitate the behavioral recovery.

H_3_Rs binding changes observed in the TMN at both stages of vestibular compensation could regulate the activity of these nuclei. Indeed, it has been shown that activation of H_3_ receptors on TMN neurons inhibits multiple high-threshold calcium channels (Takeshita et al., 1998) leading to an inhibition of their firing rate (Haas, 1992). The bilateral decrease of the H_3_Rs observed in these nuclei would activate their firing rate, inducing probably the up-regulation of HDC mRNA expression observed after UVN at these two stages (Tighilet et al., 2006). Bilateral down-regulation of H_3_R located on terminals of histaminergic TMN neurons that synapse with other TMN neurons should increase the HA synthesis and release.

### HISTAMINERGIC SYSTEM PLASTICITY IN THE IO AND THE SN

The IO subdivisions showed H_3_ binding changes similar to that observed in the VN and the PH. The olivo-cerebellar projections are known to be indispensable for vestibular compensation. Indeed, electrolytic and chemical lesions of the IO prevents vestibular compensation and causes reappearance of UL symptoms after the vestibular compensation has been established (Llinas et al., 1975). It has also been reported that UL or UVN induces expression of plasticity markers such immediate early genes (Kaufman et al., 1992; Cirelli et al., 1996; Sato et al., 1997; Gustave Dit Duflo et al., 1999) and Brain Derived Neurotrophic Factor gene (Li et al., 2001) in the IO. The down-regulation of H_3_ receptor binding sites observed unilaterally at 1 week and bilaterally at 3 weeks in the different subdivisions of the IO could be the result of an increased histamine release originating from the TMN. Functionally, by its action on IO neurons Histamine could reorganize both the olivo-vestibular and the olivo-cerebellar systems involved in the oculomotor and the postural recovery.

Concerning the vestibulo-solitary pathways, both anatomical and electrophysiological studies have shown that the solitary nucleus receives input from the vestibular nuclei that participate in vestibulo-sympathetic reflexes (Yates et al., 1994). Since the SM receives dense gastrointestinal input (Norgren, 1978; Leslie et al., 1982; Shapiro and Miselis, 1985), H_3_Rs binding asymmetry observed in this nucleus 1 week after UVN may reflect the increased salivation, retching, and emesis which are present at this post-lesional delay.

### PHARMACOLOGICAL IMPLICATIONS

Whether H3 receptors plasticity plays a significant role in the recovery process is a question of interest for a better understanding of vestibular compensation and for pharmacological applications to vestibular pathology. HA has been largely used for treatment of vertigo and disturbances of the inner ear assumed to be of vascular origin (Fischer, 1991). Betahistine is a structural analog of HA that is effective also in vestibular syndromes unrelated to vascular insufficiency like peripheral vestibular disorders (Canty and Valentine, 1981; Oosterveld, 1984) and Menière’s disease (Frew and Menon, 1976; Bertrand, 1982). We previously showed that behavioral recovery after UVN in our cat model was strongly accelerated by betahistine (Tighilet et al., 1995). We also demonstrated that this drug induced an up-regulation of HDC mRNA in the TMN and a reduction of [^3^H]N-α-methylhistamine labeling in both the TMN, the VN complex, and the three IO subnuclei (Tighilet et al., 2002). Hence, vestibular lesion as well as treatment with a structural HA analog induce similar plastic changes of the histaminergic system (H_3_R), strengthening the hypothesis that HA may elaborate and maintain the vestibular compensation process. Taken together, our results also point to the potential interest of compounds like H_3_Rs antagonists or inverse agonists (Morisset et al., 2000) as anti-vertigo drugs.

In conclusion, our study shows that UVN induces robust changes in H_3_ receptors binding at the different stages of the vestibular compensation in the cat. These changes are observed not only in the VN but also in other central nervous system (CNS) structures such the PH, the TMN, and the IO supporting the view of Llinas and Walton (1979) that vestibular compensation is a distributed property of the CNS. This result strengthens the hypothesis that histamine could be a preferential candidate in the elaboration and the maintenance of vestibular compensation process. The specific target of histamine in vestibular recovery is the H_3_ auto and/or heteroreceptors located on different brain structures, including the VN. This H_3_R target would additionally lead to positive side effects on the behavioral recovery by increasing the vigilance level and improving post-lesion sensorimotor activity and cognitive functions (Brioni et al., 2011).

## Conflict of Interest Statement

The authors declare that the research was conducted in the absence of any commercial or financial relationships that could be construed as a potential conflict of interest.
